# The Great British Medalists Project: A Review of Current Knowledge on the Development of the World’s Best Sporting Talent

**DOI:** 10.1007/s40279-016-0476-2

**Published:** 2016-02-03

**Authors:** Tim Rees, Lew Hardy, Arne Güllich, Bruce Abernethy, Jean Côté, Tim Woodman, Hugh Montgomery, Stewart Laing, Chelsea Warr

**Affiliations:** 1 Department of Sport and Physical Activity, Faculty of Management, Bournemouth University, Dorset House, Talbot Campus, Fern Barrow, Poole, BH12 5BB UK; 2 Sport, Health and Exercise Sciences, Bangor University, George Building, Bangor, Gwynedd, LL57 2PZ UK; 3 Department of Sport Science, University of Kaiserslautern, Erwin Schrödinger Street, 67663 Kaiserslautern, Germany; 4 School of Human Movement and Nutrition Sciences, Faculty of Health and Behavioral Sciences, The University of Queensland, St Lucia, QLD 4072 Australia; 5 School of Kinesiology and Health Studies, SKHS Building 28 Division Street, Queen’s University, Kingston, ON K7L 3N Canada; 6 School of Life and Medical Sciences, University College London, Rockefeller Building, 20, University Street, London, WC1E 6DE UK; 7 UK Sport, 21 Bloomsbury Street, London, WC1B 3HF UK

## Abstract

The literature base regarding the development of sporting talent is extensive, and includes empirical articles, reviews, position papers, academic books, governing body documents, popular books, unpublished theses and anecdotal evidence, and contains numerous models of talent development. With such a varied body of work, the task for researchers, practitioners and policy makers of generating a clear understanding of what is known and what is thought to be true regarding the development of sporting talent is particularly challenging. Drawing on a wide array of expertise, we address this challenge by avoiding adherence to any specific model or area and by providing a reasoned review across three key overarching topics: (a) the performer; (b) the environment; and (c) practice and training. Within each topic sub-section, we review and calibrate evidence by performance level of the samples. We then conclude each sub-section with a brief summary, a rating of the quality of evidence, a recommendation for practice and suggestions for future research. These serve to highlight both our current level of understanding and our level of confidence in providing practice recommendations, but also point to a need for future studies that could offer evidence regarding the complex interactions that almost certainly exist across domains.

## Key Points

**Table:** 

We identify what is known and what is thought likely to be true in relation to understanding the development of the world’s best sporting talent, make recommendations for policy makers and practitioners to act on, and suggest fruitful avenues for future research.
Examining topics related to the performer, the environment, and practice and training, our analysis highlights variation in the quality of evidence relevant to the development of the world’s best sporting talent, such that the strength of evidence in some topics (e.g. anthropometric and physiological factors) is higher than in others (e.g. birthdate).
We provide an authoritative, balanced, comprehensive, fully referenced and critical review of the literature, which should serve as a key point of reference (a) for researchers in talent identification and development in sport, as well as a guide to future research; and (b) for practitioners and policy makers in sport seeking an overarching, evidence-based understanding of the current state of knowledge in the area, as well as a guide for translating that knowledge into action.

## Introduction

With the competition for medals at Olympics and World Championships intensifying, there is greater investment than ever in sporting systems and structures to identify and develop exceptionally talented athletes. The Australian Institute of Sport has been credited with boosting Australia’s medal haul from five medals in the 1976 Montreal Olympics to 60 medals in the 2000 Sydney Olympics. Team Great Britain (GB)’s fourth position in the 2008 Beijing Olympics medals table was supported by a markedly increased investment (£235M), and this funding continued to support Team GB’s climb to third position in the 2012 London Olympics (£261M). When organizations such as UK Sport (the UK’s high performance sports agency) commit a further £355M of public funds to the Rio 2016 Olympic cycle, it becomes increasingly necessary to be able to draw on an evidence-based understanding of the identification and development of the world’s best sporting talent to maintain the success that is expected with this expenditure. This is the context for the present review, which seeks to identify what is known and what is thought likely to be true in relation to understanding the development of the world’s best sporting talent.

In September 2009, UK Sport invited all UK academic institutions to submit tenders to (a) “research and understand elements of identification and development, to ultimately inform the prediction of future elite sporting talent”; and (b) “conclude unique recommendations from the research that highlight key accelerants and retardants in the pathway development of elite performers”. As part of the subsequent work, the research team (led by the two first authors) drew together a panel of international research experts, elite athletes, coaches from the GB World Class Programme and expertise from UK Sport’s Research and Innovation, and Athlete Development disciplines, and UK Sport’s Senior Management Team. An initial series of meetings, presentations and workshops was held between April 2010 and January 2011. The topics highlighted and conclusions drawn from these sessions guided the development of a review/position statement with regard to current understanding of the performance and development of ‘super-elite athletes’. This review/position statement was used both in strategic planning for Rio 2016 (in March 2013) and to inform a separate research study which further explored the development of super-elite athletes. This process provided the initial guide for the present article, which was subsequently further revised and up-dated through 2015. Of particular note was the first meeting of the collaborative team at UK Sport’s headquarters in Loughborough, UK in June 2010. At this meeting, contributors were asked to present on their key topic(s) of expertise and calibrate evidence in relation to non-elite, junior elite, elite and super-elite levels of performance.

There is a voluminous literature devoted to understanding the development of sporting talent. In addition to numerous peer-reviewed journal articles, there are various academic books [1–4], reviews/position papers [5–16], governing body documents (e.g. from the UK, the USA and Australia [17–25]), popular books [26–32], specific models of talent development [33–45] and other related works of note [46–55]. With so much information and opinion across many sub-disciplines of the sports sciences, so many models and frameworks, so many levels of performer, such varied levels of empirical knowledge and much apparent truth, popular wisdom and controversy, the task of generating a clear understanding of the development of the world’s best sporting talent was challenging.

In order to provide recommendations for best practice in which readers could have confidence, we believed it was important to move beyond a purely narrative description of research evidence to rate the quality of evidence available. Thus, we provide additional information, by focusing on three key aspects:

- Categorization of the performance level of the study samples as *non-elite* (juniors or seniors competing below national level), *junior elite* (junior national to junior international level), *elite* (senior international level) or *super-elite* (Gold medalists at Olympics or World Championships);^1^
- Employing a modification to the GRADE (Grading of Recommendations Assessment, Development and Evaluation) system [56] to rate the quality of evidence (based on study design, consistency of evidence and directness of evidence)—indicating the extent to which we can be confident that an estimate of effect is correct;^2^^,^^3^ and
- Offering a recommendation (as noted in the GRADE [57] guidelines) to policy makers and/or practitioners on whether to draw on the evidence and use it in practice.

We should stress that a strong body of evidence does not necessarily in itself lead to a strong recommendation for practice. For example, one may make confident recommendations when the quality of evidence is high, but the potential benefits of applying knowledge may only lead to modest practical gains. Conversely, one may be less confident in making recommendations because the quality of evidence is low or moderate, yet the potential benefits may be compelling.

## The Performer

### Birthdate

Athletic success may be influenced by birthdate. The *relative age effect* (RAE) refers to a biased distribution of elite athletes’ birthdates, with an over-representation of those born at the beginning of any given competitive year (e.g. September in most Western societies) and an under-representation of those born at the end (e.g. August). A meta-analysis [58] of studies from 1984 to 2007, examining non-elite-, junior elite- and elite-level athletes showed robust support for the RAE across ice hockey, soccer, baseball, basketball and volleyball. More recent research with junior elite samples [59–61] has provided additional evidence for this RAE with ice hockey, handball and soccer.

Although the above results appear convincing, there is evidence that RAEs may be inconsistent. In some elite samples [62–65], RAEs have been demonstrated in ice hockey and baseball, but not in American Football, basketball, soccer, golf, handball, taekwondo, volleyball and various other unspecified Olympic sports. Elite-level ice hockey players [66] have demonstrated moderate evidence for RAEs in ‘average’ players, but a reversal of the effect in the ‘most’ elite players (‘All Star’ and Olympians), with relatively younger players enjoying longer careers. There is also evidence [67] at junior elite level that RAEs may be more prominent in boys than in girls, as well as evidence that younger athletes figure more prominently in earlier rounds of drafting into US National Hockey teams, and some elite-level data [68] demonstrating a greater proportion of relatively younger players at later stages of careers.

Research [69, 70] with non-elite- and elite-level samples has cautioned against the normal comparison of observed birthdates with an expected distribution of birthdates, because the distribution of birthdates within sports may be uneven due to younger athletes not choosing a particular sport—a form of ‘self-restriction’—and younger athletes being more likely to drop out. For example, when the population of soccer players originally registered in the sport was taken as the comparison group, the RAE disappeared [70].

With *moderate* study design, *low* consistency and *moderate* direct evidence (up to elite level), the quality of the evidence that being relatively older is an advantage with regard to the development of super-elite performance in sport is *moderate to low*. The evidence suggests that any advantage associated with being born in the first two quarters of the year may disappear by the time athletes reach elite level. We therefore *recommend* that practitioners do *not* make use of RAEs for talent selection [71] or development purposes, but rather policy makers and practitioners focus on structuring the environment to limit the negative effect of relative age [72, 73]. More research is needed to better understand the extent to which RAE-related effects occur (a) at initial involvement in a sport (‘self-selection’), (b) after prolonged involvement in competitions (success-related selection), or (c) on selection to an athlete support programme (explicit selection). Researchers should also carefully consider the most appropriate comparator groups. Further, it would be helpful to examine the extent to which the competitive level and range of sports available throughout development is responsible for the RAE—that is, with more players and fewer available sports are RAEs more pronounced?

### Genetics

It would appear no longer a case of whether there is a genetic component to sporting performance, but rather which genetic profiles make the greatest contribution [74]. There is evidence at non-elite level [75–79] that genetic factors explain 20–80 % of the variance in a host of measures: explosive strength, speed of limb movement, running speed, reaction time, flexibility, balance, bone mineral density, lean muscle mass, eccentric arm flexor strength, concentric arm flexor strength, arm cross-sectional area, change in maximum voluntary force, isometric strength and *V*O_2_max. Specific gene variants appear to influence participation in physical activity [80]—the GENEATHLETE project claims to have identified a phenotype for athletic status by comparing athletic samples with sedentary people [81, 82]. Indeed, 66 % of the variance in non-elite ‘athlete status’ may be explained by genetic factors [83].

A significant heritable component has been identified with non-elite samples in agility, sprinting, jumping, throwing, kinematics and reaction time [76, 84–86], and also in personality/character [87]. Specific gene variants may influence the determination of endurance/aerobic and muscle strength/anaerobic performance [88–91]. In particular, substantial attention has been paid to the relationship of ACTN3 (actinin alpha 3) and VDR (vitamin D receptor) gene variants with strength/power. Within the ACE (angiotensin I-converting enzyme) gene, the absence (deletion, D allele) rather than presence (insertion, I allele) of 287 base pairs is associated with higher circulating ACE activity [92, 93]. The I allele is generally associated with fatigue resistance and endurance performance, and the D allele with power/strength/sprint phenotypes [94, 95]. The I allele is associated with training-related improvements in loaded repetitive biceps performance [96], its frequency is increased among elite-level high altitude mountain climbers [96] when compared with controls, and it is associated with success in summiting even among non-elite samples [97, 98]. I allele frequency rises with distance run in elite-level runners [99]. Conversely, the D allele is associated with sprint/power performance in elite short-distance swimming [100–102].

Genetics are also related to susceptibility to injury [103]. The E4 variant of the apolipoprotein E epsilon4 (ApoE4) may be associated with increased severity of chronic neurological deficits in high-exposure non-elite boxers [104], while genetic variation within the collagen type 5 alpha 1 (COL5A1) gene has been associated with Achilles tendon [105] and anterior cruciate ligament injury [106] in non-elite athletes when compared with non-injured controls. The field of epigenetics [107–110] offers evidence of (heritable but reversible) changes in gene expression, which do not involve a change in the DNA sequence (i.e. gene expression may result from environmental influences). The fact, for example, that mothers’ activity levels might influence gene expression (across generations) could likely have important implications for the emergence of sporting talent. Work on (functional) genomics [111–113] has demonstrated compelling evidence of changes in gene expression relating to functional adaptation in response to muscle activity in endurance training.

With *high* study designs, *moderate* consistency and *moderate* direct evidence (up to elite level), the quality of the evidence that genetics could make an important contribution to talent selection and development in sport is at least *moderate*. Indeed, although rare combinations of gene variants are likely to act in concert to yield propensity to super-elite athlete status [114], and elite performance cannot necessarily be predicted well from genetic factors, genetic factors may influence the sport in which athletes are most likely to successfully compete [115]. Genetic selection methodologies may, however, come with negative reputational, personal, ethical and societal impacts. We therefore *recommend* that policy makers and practitioners consider the possibility of using genetic profiling to help athletes make more informed and appropriate decisions about sport type and discipline during their development years. We may only be able to evaluate the true benefits of genetic testing when geneticists and sports scientists collaborate in large prospective cohort studies that empirically determine the utility of genetic analyses in predicting future performance. The potential impact of genetics could be great, and thus further research in this area is warranted, in particular in relation to specific performance genes, training/learning genes and genes underpinning injury proneness.

### Anthropometric and Physiological Factors

There is a long history of anthropometric studies of Olympic athletes, dating back to documenting the physique of track and field athletes at the 1960 Rome Olympics [116]. As a result, both anthropometric and physiological factors have now been identified across a number of sports at all levels of performance: non-elite [117–120], junior elite [121–128], elite [129–131] and super-elite [132]. Among the many variables examined are: height, weight and (lean) body mass; bone mineral content and density; limb length and circumference; amount of adipose tissue; jumping and sprinting ability; strength; and *V*O_2_max. This research has examined a wide range of sports, including: Australian Rules football, basketball, canoe polo, field hockey, football, handball, netball, rowing, rugby league and tennis. Clearly, aerobic capacity, anaerobic endurance and anaerobic power [133] are important for optimal sport performance, with a large proportion of training focused on these qualities, and with specific protocols for physiological assessments likely to be different across different sports [134, 135].

Although morphology-related factors may be involved in directing some athletes to specific sports [136]—e.g. gymnasts and divers are typically the smallest and lightest of all athletes; weightlifters and powerlifters have a high ratio of sitting height to stature caused by shorter than average upper and lower limb lengths—some argue [11] that anthropometric research has been over-interpreted, leading to the questionable practice of anthropmetric profiling to identify athletes for early selection and specialization in a sport. Factors such as individual variability in growth, the unstable nature of anthropometric—as well as physiological—measures throughout adolescence and the limited predictability of performance potentially limit the utility of anthropometric and physiological measures for talent identification purposes. Biological maturation should thus be accounted for in talent identification [123, 137]. Hormonal changes during puberty result in physical and physiological changes, which are important for sporting performance. A review [138] across many sports with non-elite and junior elite data concluded that significant changes during puberty make the prediction of adult performance from adolescent data challenging.

With *high* study design, *high* consistency and *high* direct relevance (up to super-elite level), the quality of the evidence that anthropometric and physiological factors contribute to the development of super-elite performance in sport is *high*. However, changes during puberty make the prediction of adult performance from adolescent data unreliable. We therefore *recommend* that practitioners make use of physiological testing for purposes of informing the training process, and make use of anthropometric profiling and physiological tests for both talent selection and development purposes, but policy makers and practitioners should ensure that such action is accompanied by appropriate procedures (considering biological maturation) to ‘re-capture’ lost/missed late maturers. The most obvious issue for talent identification researchers in sport to solve is the problem of predicting adult performance from adolescent anthropometric and physiological data. Solving this conundrum could have an enormous impact on talent identification procedures.

### Psychological Skills and Motivational Orientations

As long ago as 1977, Mahoney and Avener [139] attempted to identify some of the psychological characteristics of elite gymnasts. There is now evidence at non-elite [140–146], junior elite [147–149], elite [23, 139, 150–156] and super-elite [151, 157–164] level that more successful athletes display higher levels of motivation, higher levels of confidence and perceived control, higher levels of mental toughness and resilience, better ability to cope with adversity, greater resistance to ‘choking’ (i.e. performing worse than expected [165, 166]) in high-pressure situations, and command a wide range of mental skills (e.g. goal-setting, anxiety control, imagery, self-talk and decision-making).

Evidence at elite [23, 153] and super-elite [157, 161, 163, 164] level suggests that athletes display a strong task orientation to base their perceptions of competence on personal improvements, but that at non-elite [167], junior elite [168], elite [169] and super-elite [163, 170] level athletes also display a strong ego orientation to formulate perceptions of competence by comparing their own ability with that of others. There is also evidence that non-elite- [171, 172] and elite- [173] level athletes can use anxiety to enhance their performance. In particular, athletes have been noted to produce both their best and their worst performances when anxious [172]. This may be because anxiety is associated with higher levels of effort [171, 174], which could lead to higher levels of performance, provided the performer does not lapse into attempting to consciously control each specific movement or action [166, 175, 176]. Higher performing athletes also interpret their anxiety symptoms as being more facilitative to their performance than lower performing athletes [177, 178].

There is evidence at non-elite and elite level [179–181] that successful athletes display self-determined forms of motivation, and that the greater the levels of this form of motivation, the lower the risk of burnout. However, there is also evidence that elite athletes have higher levels of extrinsic motivation and lower levels of intrinsic motivation than less accomplished athletes [182, 183]. More recent research [184] has found that obsessive (more controlling) passion in non-elite athletes is a stronger predictor of deliberate practice (see Sect. 4.1), and thus sports performance, than harmonious (more self-determined) passion.

With *moderate* study design, *high* consistency and *high* direct relevance (up to super-elite level), the quality of the evidence that psychological factors are an important contributor to the development of super-elite performance in sport is *high to moderate*, although the evidence is more widespread across some psychological characteristics than others. We therefore *recommend* that practitioners make use of psychological profiling for talent development purposes. Key questions for future research include examining the *causes* of exceptional levels of motivation, resilience and mental toughness, including assessing whether and how psychological skills at junior level influence long-term adult elite/super-elite performance. How do exceptional performers use their anxiety in a positive way? How do the world’s best performers maintain focus and concentration, while avoiding lapses into conscious control? How can these skills be trained?

### Personality Traits

There is evidence at non-elite [185–188], elite [23, 189] and super-elite [157, 161, 164] level that more successful athletes display greater conscientiousness, dispositional optimism and hope than less successful athletes. There is also evidence at non-elite [190–192], elite [23, 164] and super-elite level [161] that athletes display *adaptive* perfectionism—a tendency to maintain perspective on performances while striving to achieve exceptional standards. This contrasts with the many negative outcomes (e.g. burnout, preoccupation with mistakes and self-doubts) associated with (maladaptive) perfectionism [193]. There is evidence at non-elite level [194–198] for the influence of *narcissism* on performance. Narcissists have an inherent (albeit unrealistic) belief in their ability [199], but this self-belief may well facilitate very high levels of performance under pressure [198].

With *moderate* study design, *moderate to low* consistency (generally consistent, though relatively infrequent) and *high* direct relevance (including super-elite level), the quality of the evidence that personality is an important contributor to the development of super-elite performance in sport is *moderate*. Furthermore, the risks associated with practitioners acting on the available evidence for talent development purposes seem to be only modest, although the same cannot be said with regard to using it for talent selection purposes. We therefore *recommend* that practitioners might make use of personality profiling for talent development but not talent selection purposes. Future research could focus on whether there are other important (combinations of) personality characteristics that are necessary for the development of a strong competitive personality and how these characteristics might be best developed.

## The Environment

### Birthplace

There is evidence across junior elite [59, 200, 201] and elite levels [64, 65, 202–204] that the size of the city where an athlete spends his/her developmental years can affect the likelihood of attaining elite-level performance. Small- to medium-sized communities (circa 30,000–1,000,000) appear to offer the greatest opportunities for success, although there is wide variation (not least because a medium-sized city in one country may be considered small or large in another), and in UK-based data [63], areas with populations of 10,000 and 29,999 are more likely to produce Olympic athletes, with areas between 500,000 and 999,999 being disadvantaged. A birthplace effect analysis [205] with elite and super-elite athletes from the UK World Class Programme (WCP) revealed the following: Compared to the general UK population, WCP athletes were 2.1 times more likely to be born in a medium-sized town (50,000–99,999 residents), 10.5 times more likely to attend a primary school in a very small village (<1999 residents) and 3.0 times more likely to attend secondary school in a very small village (<1999 residents). Birthplace itself may not be as critical as place of early development. Indeed, birthplace effects may be buffered by broader psychological, social, structural and cultural mechanisms [63, 200, 204, 206]. Nevertheless, birthplace effects provide support for the notion that environments vary in their capacity to develop sporting talent and that ‘talent hotspots’ may be a reality.

With *moderate* study design, *high* consistency and *high* direct relevance (up to super-elite level), the quality of the evidence that birthplace offers an advantage with regard to the development of super-elite performance in sport is *high to moderate*. We therefore *recommend* that policy makers and practitioners at least take consideration of birthplace when designing talent search initiatives as well as profiling athletes during talent selection and development. Understanding more about the physical and social environment, organisation of resources and the number of participants competing for available places in sports are key areas for research—i.e. understanding more about the environments and neighbourhoods that potential sporting talents are exposed to, and less about birthplace population size.

### Support from Parents, Family, Siblings and Coaches

The importance of family and siblings during athletes’ developmental years has been highlighted [39]. Evidence from non-elite [207–216], junior elite [148, 217–219], elite [23, 220–222] and super-elite [158–160] athletes attests to the influence of social groups, social support and support networks [223] (including family, coaches, other athletes/peers and support staff). In addition to their key role in the provision of expert coaching and training, coaches can help to enhance the development of psychological skills and mental toughness in athletes during their developmental years [50, 158, 161, 224, 225]. Non-elite data [226, 227] suggest that the supportiveness and feedback effectiveness of coaches is dependent on a unique fit (and common identity) between the characteristics of the coach and the personality of the athlete.

With *moderate* study design, *moderate* consistency and *high* direct relevance (up to super-elite level), the quality of the evidence that support plays a role in the development of super-elite performance in sport is *at least moderate.* We therefore *recommend* that policy makers and practitioners heed the important influence of the support process during talent development. However, it is worthy of note that the nuances of providing appropriate support appear to be much more complex than most lay people realize. There is still a relative lack of knowledge with regard to the influence of early family experiences, and we need to know more about the role of the family (parents, siblings, inter-relations) more generally with respect to who reaches super-elite level in sport.

### Athlete Support Programmes

Evidence from 19 European countries [228] suggests that most talent identification systems in sport use current junior performance and/or early competitive success as the main criterion for selection to a development programme. Although most elite and super-elite athletes have been involved in athlete support programmes at some stage [20, 229], there is evidence across all performance levels [13, 20, 228–234] that junior success does not significantly predict long-term senior success. A 7-year longitudinal study of 4686 German athletes (from athletics, cycling, field hockey, rowing, table tennis, weight lifting and wrestling) across all performance levels [229] and a 12-year longitudinal study involving 1420 members of 13 soccer academies [235] revealed: (a) considerable annual turnover of athletes within each squad; (b) the younger the first recruitment to a support programme, the younger the exit from the programme; and (c) the higher the attained level within an athlete support programme and the higher the level of senior success, the later the age of first recruitment. Various other studies have highlighted super-elite performers being recruited to support programmes at significantly later ages than their elite counterparts [228, 229, 236, 237]. Interestingly, UK data [13] suggest that athletes selected via ‘Talent Transfer’ programmes at ages 16–25 years can reach the performance of their elite peers within 1 year. Relatedly, data from German elite and super-elite athletes at the Summer 2004 and Winter 2006 Olympic Games [238] and from Dutch non-elite and elite athletes [239] reveal no differences in medal success between athletes who attended “elite sport schools” and those who did not, while the latter attained higher academic achievements.

With *moderate* study design, *moderate to low* consistency (i.e. consistent but infrequent), and *high* direct relevance (up to super-elite level), the quality of the evidence regarding early athlete support programmes’ contribution to the development of super-elite performance in sport is *moderate.* The trajectory to super-elite status appears distinctly non-linear [240], involving repeated selection and de-selection, rather than linear progression within athlete support programmes [235]. We therefore *recommend* that policy makers and practitioners appreciate that junior success does not contribute significantly to predicting long-term senior success, that early athlete support programmes are not the sole route to the development of talent, that support programmes be open for access at all age ranges, and thus that de-selected athletes be monitored for potential return. Empirical evaluations of the efficacy of athlete support programmes would appear a priority for future research, with the potential to encourage a major re-think of some of the components of the current support programme strategy. At a more theoretical level, why do some apparently ‘talented’, highly motivated athletes fail to progress at key transition points (especially from junior elite to elite level)? Do sport systems typically require the talented athlete to ‘fit in’ more than they adapt to allow the athlete to thrive toward excellence?

## Practice and Training

### Volume of Sport-Specific Practice and Training

Despite wide variation across sports, most junior elite, elite and super-elite athletes have accumulated enormous volumes of organized practice and training [149, 230, 241–260]. Extensive sport-specific deliberate practice (DP) is thus a pre-requisite to world-class performance in sports with a large participant base.

A widely held view, based on seminal work in chess [261] and music [262], is that 10 years and 10,000 h of DP are necessary and sufficient to reach expert level [27, 31]. Indeed, many elite and super-elite athletes have been practicing and training for ten years or longer [230, 241, 243–245, 248]. In discussing his DP framework, however, Ericsson [263] has recently emphasized he did not intend for his original (i.e. 1993 [262]) conclusions to constitute a 10,000 h ‘rule’. In fact, there is considerable variation within and across sports at elite and super-elite level [10, 23, 264], with some data suggesting an average time from novice to senior national representation of just 7.5 years, and even Olympic level in just 14 months [265]. Evidence at super-elite level suggests as few as 4400 h may lead to Olympic Gold in field hockey [236], and 4500 h to representing the German national soccer team [266], with just 4000 h sufficient to reach elite and super-elite levels in basketball, field hockey and netball [241]. Interestingly, organized practice/training has been shown at junior elite [267] and super-elite level [266] to comprise considerable non-DP activity (e.g. play).

DP theory also asserts [262] that the more DP accumulated, the higher the performance attained. There is evidence that more successful athletes have averaged larger amounts of organized sport-specific practice/training. These observations are based on comparisons of non-elite athletes with: junior elite athletes in cricket and soccer [246, 259, 260]; elite athletes in basketball, cricket, field hockey, handball, soccer, swimming, triathlon and wrestling [243, 248, 250, 255, 260]; and super-elite athletes in basketball, darts, field hockey and netball [241, 245], and also on comparisons of adolescent elite with super-elite rhythmic gymnasts [254]. Additionally, elite Australian Rules footballers with better perceptual/decision-making skills performed more domain-specific practice than less skilled players [244]. Differences in the amounts of organized domain-specific practice/training were, however, only significant in these studies for data referring to training in late adolescence and adulthood, not training at younger ages (except rhythmic gymnastics [254]).

Such differences have not been observed between super-elite and elite athletes in field hockey, soccer, tennis, swimming [203, 236, 253, 266] and across all Olympic sports (including athletics, badminton, basketball, fencing, figure skating, gymnastics, judo, rowing, soccer, swimming, table tennis and wrestling) [20, 230]. The opposite effect has also been noted, with super-elite field hockey players training significantly less than their elite peers [258]. No consistent differences have been reported with regard to the volume of competition experienced between different success levels [241, 244–246, 259].

Although the DP framework has gained popularity in sport science and in popular literature, its applicability to high-performance sport may be limited. The suggestion of 10 years/10,000 h was originally based on: (a) musicians, not outstanding athletes; and (b) a strict interpretation of DP, excluding intrinsically enjoyable activities, team practice, play, competition, non-organized sporting activities, and also ruling out implicit (improved task performance in the absence of conscious awareness) and incidental learning (learning in the absence of an intention to learn). DP also implies full attention and concentration, while research indicates that full concentration does not always generate optimal learning/performance. Increasing conscious awareness may even result in poorer performance (e.g. paralysis by analysis [268]; the regression hypothesis [176]—(i.e., regressing to a performance level akin to earlier learning). Evidence at non-elite level [176, 269] also indicates that implicit learning leads to more robust performance under pressure. Finally, evidence at junior elite [246, 259, 260, 270], elite [242, 244] and super-elite level [241, 245, 266] demonstrates that organized and non-organized play is an important component of (early) experiences of developing sporting experts.

With *moderate/high* study design, *moderate* consistency and *high* direct relevance (up to super-elite level), the quality of evidence that extensive DP is an important contributor to the development of super-elite performance in sport is *high* to *moderate*, while *high/moderate* quality of evidence suggests that the applicability of the 10 years/10,000 h rule is limited and that DP alone does not guarantee sporting success. Additionally, the contribution of practice/training to the development of sporting expertise may only apply to domain-specific practice accrued during late adolescence or adulthood, with practice volume not discriminating elite from super-elite athletes. Finally, there is some *low* quality evidence to suggest that automaticity and implicit learning may contribute to the development of sporting expertise. We therefore *recommend* that policy makers and practitioners continue to promote deliberate practice, but consider the present evidence before routinely increasing practice volumes with junior athletes, and acknowledge the potential benefits of automaticity, implicit learning and also enjoyment in practice and play. The links between early sport-specific practice/training and short- and long-term outcomes are a research priority. How is intensified specific training related to long-term enjoyment, motivation, stress-recovery and prolonged involvement? Future research should also further explore the roles of explicit and implicit/incidental learning in the development of expert performance. This implies scrutiny of the intentions and specific activities performed during practice/training and play, their combinations, variability, potential interactions and relative influence through different developmental age ranges.

### Early Specialization Versus Sampling and Play

Where peak performance in sport is achieved before biological maturity, early specialization may be necessary to reach elite level. For example, super-elite athletes in artistic composition sports (artistic gymnastics, figure skating, platform diving and rhythmic gymnastics [230]) performed three to seven times more sport-specific training until age 10 years compared to all other types of Olympic sports. Their volumes of practice/training did not, however, differ from their elite counterparts *within* their respective sports. A super-elite sample of rhythmic gymnasts also experienced reduced involvement in other sports compared to their elite counterparts [254]. However, evidence at non-elite, junior elite, elite and super-elite level suggests that many athletes have not progressed exclusively within one discipline, but have practiced multiple sports during childhood and adolescence [230, 236, 241–244, 260, 270–274]. Further, evidence from non-elite and super-elite data [254, 275–278] points to the potential costs and risks associated with early specific practice, training and competitions (e.g. less enjoyment, time demands, restricted activities outside sport, exhaustion, overuse injuries and increased risk of dropout). Comparisons between super-elite and elite athletes from field hockey, soccer, tennis and 47 Olympic sports [203, 230, 236, 266] have even demonstrated larger volumes of practice/training and/or play in other sports among the super-elite, mostly associated with a later start in their main sport and a later specialization.

There is also evidence at non-elite, junior elite, elite and super-elite level that many athletes have spent considerable time in non-organized play during childhood [39, 241, 244, 246, 255, 259, 260, 270]. A positive relationship between non-organized play and junior elite [246, 260] and super-elite success [266] has been noted, but equally other studies have noted no differences between performance levels, with some demonstrating more play among non-elite compared with elite/super-elite athletes [236, 241, 244, 245, 255, 259, 260]. Elite and non-elite soccer players [279] could be differentiated by a *combination* of above-average volume of organized soccer training/practice with *either* above-average involvement in other sports *or* above-average non-organized soccer play.

With *moderate* study design, *moderate* (early sampling of diverse sports, late specialization)*/low* (play) consistency, and *high* direct relevance (up to super-elite level), the quality of the evidence that early specialization or sampling represent the best route to the development of super-elite performance in sport is *moderate.* Both early specialization and sampling (and play) may be routes to expertise under optimal conditions. However, the probability of attaining elite or super-elite level may be enhanced by the *coupling* of a large volume of intensive, organized specific training/practice in the main sport with appreciable amounts of organized training/practice and competitions in other sports and/or non-organized play in the main or other sports. We thus *recommend* policy makers and practitioners to draw on this evidence, bearing in mind the need to minimize the potential hazards of early specialization when such specialization is necessary, and with regard to promoting opportunities for young athletes to experience non-organized play and sampling in a variety of sports. Future research is needed to understand how participation in various sports benefits super-elite performance in one main sport. Further, how does the process of late specialization following prior diversification or ‘talent transfer’ proceed? Are there certain sports or clusters that lay the best foundation for super-elite success in a final sport?

## Other Potential Factors

There are a number of additional topics that have been raised in the literature, which do not meet the level of evidence of the other topics in this review (e.g. descriptive, anecdotal, non-elite sport or one study). Under-studied in sport, the quality of evidence for these topics is thus low, and we cannot make recommendations to act. Nonetheless, they may still be intriguing ‘candidates’ for future examination. They include: the role of the family’s socioeconomic status in different sports and countries [203, 280–282]; the different routes to super-elite level across cultures [237]; making errors in the learning process without penalties or consequences [283]; the significance of recovery, rest and sleep to optimize the benefits of practice [284–287], potentially linked to the reminiscence effect (i.e. ‘improvement in the performance of a partially learned act that occurs while the subject is resting’ p. 3; [288]); the opportunity in sport for athletes to identify, express and (thereby) exercise control over their emotions, which in normal life they find difficult to express [289, 290]; and finally, a potential impact of childhood emotional trauma on qualities such as mental toughness, grit, resilience, growth mindset, achievement striving and ability to overcome difficulties [291–293]—and relatedly, positive or negative ‘critical’ events with high personal significance (e.g. success milestones, squad selection, non-selection, losses, injury, school disruption, parental divorce and bereavement [23, 156, 158, 159, 291, 294–296]).

## Conclusion

We reviewed key topics (see Table 1) relevant to the development of the world’s best sporting talent, generating a current level of understanding, recommendations to act and suggestions for future research. In encouraging researchers, we would point to the relative dearth of prospective and multidisciplinary studies that could offer insight regarding the complex interactions that almost certainly exist across domains. Embracing this complexity remains the most obvious future direction.

**Table 1:** Overview of research into the development of the world’s best sporting talent: study design quality, consistency of evidence, directness of evidence and key points

Topic	Study design quality	Consistency of evidence	Directness of evidence
The performer			
Birthdate	Moderate	Low	Moderate
Relative age effects exist but may not be robust across all sports			
Genetics	High	Moderate	Moderate
Genetics may influence and thus limit the development of performance. Performance cannot, however, be well predicted from genetic factors. Caution should be urged for ethical and societal reasons when considering genetic selection methodologies			
Anthropometric and physiological factors	High	High	High
Anthropometric and physiological factors are important for performance. However, caution should be urged when using anthropometric and physiological tests for talent selection purposes with adolescents because of variation in biological maturation			
Psychological skills and motivational orientations	Moderate	High	High
Psychological factors (e.g. motivation, confidence, perceived control, mental toughness, resilience, coping with adversity, resistance to ‘choking’, mental skills) appear to be important contributors to the development of super-elite performance			
Personality traits	Moderate	Moderate/low	High
Super-elite athletes are conscientious, optimistic, hopeful and perfectionist			
The environment			
Birthplace	Moderate	High	High
Small-to-medium communities provide favourable environments for developing athletes. Talent hotspots may exist			
Support from parents, family, siblings and coaches	Moderate	Moderate	High
Super-elite athletes have benefitted from supportive families, coaches and networks during their development. The subtleties of the provision of support are not well understood			
Athlete support programmes	Moderate	Moderate/low	High
Early success is a poor predictor for later super-elite success, and thus for early talent identification purposes. Super-elite success is mostly preceded by relatively late entry into organized support programmes			
Practice, training and play			
Volume of sport-specific practice and training	High/moderate	Moderate	High
Super-elite performance develops from extensive deliberate practice, but the applicability of the 10 years/10,000 hours 'rule' to high-performance sport is limited. Play may also be relevant, as may implicit/automatic and incidental skill learning			
Early specialization vs. sampling and play	Moderate	Moderate/low	High
The key to reaching super-elite level may be involvement in diverse sports during childhood and appreciable amounts of sport-specific practice/training in late adolescence and adulthood			

## Footnotes

1 Studies of genuinely world-class (i.e. super-elite) athletes are under-represented in sport. At the same time, definitions of ‘elite’ athletes in research vary widely, from regional juniors (non-elite athletes, according to our definitions) through to Olympic gold medal winners (super-elite athletes). As well as attempting to clarify these distinctions, our reasoning for differentiating super-elite from merely elite athletes was the appreciation that there may be subtle yet fundamental differences between athletes who reach international level and those who achieve Gold at Olympic or world level. Such differences would be of great importance and relevance to sporting organizations, tasked with the role of converting elite performers into world’s best.

2 The GRADE system may be used for rating quality of evidence in reviews and guidelines and grading strength of recommendations. The system classifies the quality of evidence into one of four levels: high, moderate, low and very low. Because randomized controlled trials (regarded as the highest rating within the GRADE system) over multiple years are rarely possible in elite sport, we re-calibrated the quality and strength of research evidence by effectively inflating GRADE quality ratings by one point.

3 Our reviews were based on non-exhaustive literature reviews (using Web of Science and Google Scholar in combination with UK Sport’s archives, and the authors’ personal archives). Indeed, many unpublished reports that we considered could not have been considered in a systematic review. Thus, the conclusions are our criterion-based judgments, which we believe aligns with *Sports Medicine*’s mission to provide an authoritative, balanced, comprehensive, fully referenced and critical review of the literature.
